# Cost analysis of implementing mHealth intervention for maternal, newborn & child health care through community health workers: assessment of ReMIND program in Uttar Pradesh, India

**DOI:** 10.1186/s12884-018-2019-3

**Published:** 2018-10-03

**Authors:** Shankar Prinja, Aditi Gupta, Pankaj Bahuguna, Ruby Nimesh

**Affiliations:** School of Public Health, Post Graduate Institute of Medical Education and Research, Sector-12, Chandigarh, 160012 India

**Keywords:** mHealth, Health system cost, Costing analysis, Maternal and child health, Community health volunteers

## Abstract

**Background:**

The main intervention under ReMiND program consisted of a mobile health application which was used by community health volunteers, called ASHAs, for counselling pregnant women and nursing mothers. This program was implemented in two rural blocks in Uttar Pradesh state of India with an overall aim to increase quality of health care, thereby increasing utilization of maternal & child health services. The aim of the study was to assess annual & unit cost of ReMiND program and its scale up in UP state.

**Method and materials:**

Economic costing was done from the health system and patient’s perspectives. All resources used during designing & planning phase i.e., development of application; and implementation of the intervention, were quantified and valued. Capital costs were annualised, after assessing their average number of years for which a product could be used and accounting for its depreciation. Shared or joint costs were apportioned for the time value a resource was utilized under intervention. Annual cost of implementing ReMiND in two blocks of UP along and unit cost per pregnant woman were estimated. Scale-up cost for implementing the intervention in entire state was calculated under two scenarios – first, if no extra human resource were employed; and second, if the state government adopted the same pattern of human resource as employed under this program.

**Results:**

The annual cost for rolling out ReMiND in two blocks of district Kaushambi was INR 12.1 million (US $ 191,894). The annualised start-up cost constituted 9% of overall cost while rest of cost was attributed to implementation of the intervention. The health system program costs in ReMiND were estimated to be INR 31.4 (US $ 0.49) per capita per year and INR 1294 (US $ 20.5) per registered women. The per capita incremental cost of scale up of intervention in UP state was estimated to be INR 4.39 (US $ 0.07) when no additional supervisory staffs were added.

**Conclusion:**

The cost of scale up of ReMiND in Uttar Pradesh is 6% of annual budget for ‘reproductive and child health’ line item under state budget, and hence appears to be financially sustainable.

**Electronic supplementary material:**

The online version of this article (10.1186/s12884-018-2019-3) contains supplementary material, which is available to authorized users.

## Background

Despite the rapid improvement in maternal, newborn and child health (MNCH) indicators in India, many districts in the country still face high maternal and infant mortalities [1]. On the basis of a composite health index indicator, Government of India (GOI) identified 184 high priority districts across 29 states for focused integrated planning and monitoring of reproductive, maternal, newborn, child and adolescent health care program (RMNCH+A) [2]. District Kaushambi in Uttar Pradesh (UP) state is a high priority district with high maternal mortality ratio (MMR) of 283 per 100,000 live births and infant mortality rate (IMR) of 82 per 1000 live births [3] which are way above the national and state averages of 178 & 258 MMR and 40 & 68 IMR respectively [3]. Most of these maternal and newborn deaths are preventable if a continuum of care is followed right from pregnancy to newborn care. This includes whole spectrum of services including utilization of antenatal care, institutional delivery, early initiation of breastfeeding, essential newborn care, early recognition and referral of maternal and newborn complications with timely access to quality healthcare [4]. In order to strengthen the service provision, National Rural Health Mission (NRHM) was introduced in 2005 to augment primary health care and build capacity of community health workers (CHW) in remote areas to deliver affordable, equitable and accessible care [5]. With this aim, concept of Accredited Social Health Activist (ASHA) was envisaged. These are local women who serve as first contact between public health system and communities. ASHA as a cadre were included in the health system with intent to fulfil three roles i.e., creating awareness among communities for health issues, facilitating access to healthcare services, and as a provider for a limited range of basic health services. They help in addressing community health needs and generating demand for health service utilisation. This huge human resource of 890,000 ASHAs in the country holds immense potential and perform range of services such as mobilizing community for antenatal services during pregnancy, institutional delivery, identification and referral for maternal & newborn complications, home-based postnatal care, universal immunization, prevention of water-borne & other communicable diseases, and nutrition & sanitation [6]. With a minimal educational qualification of high school, ASHAs are provided on job training in various modules as specified by GOI [7, 8]. Various studies have been conducted to determine the effectiveness and functionality of ASHA workers in India. A study conducted in eight states of India highlighted that 75% pregnant women received services from ASHAs across all the states which included whole range of services from antenatal care, accompanying for institutional delivery, immunization etc. [9]. Findings of the 10th Common Review Mission also highlighted that coverage of antenatal care, institutional delivery and immunization services have improved in 16 states of India, which were also attributed to the role of ASHA workers [10]. Another study found that ASHA workers were valued, as service providers by rural communities in creating awareness and behaviour change towards maternal and child care [11]. However, many studies highlighted that the quality and pace of trainings provided to ASHA workers after recruitment were inadequate and service provisioning by ASHA was largely limited to health education and referral. Thus, a need for regular and refresher trainings were realised for improving ASHAs’ performance [12]. A review of the home-based care highlighted that skill retention and thus quality of care provided by ASHA could be improved by either conducting periodic refresher trainings or by using information technology, or using audio visual teaching aids that are more interactive and engaging [13]. These job aids are likely to improve retention of information and make ASHAs communication effective [13].

Mobile technology based health solutions are increasingly being utilized in health sector in low and middle income countries to strengthen skills of CHWs. Mobile Health (mHealth) as defined by World Health Organization (WHO) is an area of electronic health that provides health services and information via mobile technologies such as mobile phones and personal digital assistants [14]. For instance, mHealth application has been used for improving maternal and child health indicators in Ethiopia [15], as web based mobile applicable modules for treatment and follow up of malaria cases along Thai-Myanmar border and for community case management of malaria in Saraya, Senegal [16, 17], as diagnostic and management tool in diabetes [18] and as a cardiovascular risk assessment tool in Nyanga district of Cape town [19]. Similarly, one mobile based application was implemented as ReMiND (Reducing Maternal and Newborn Deaths) program in routine health care service delivery through ASHA workers in two blocks of district Kaushambi in UP state. The overall aim of this intervention was to increase the quality of counselling provided by ASHAs with the help of mobile application as an audio visual aid, thereby increasing utilization of MCH services.

However, a global survey done by WHO highlighted the lack of cost effectiveness data as one of major barrier in justifying implementation of mHealth services. It reported that only 13 out of the 112 countries have ever evaluated cost effectiveness of their mHealth programs [20]. Also, a recent systematic review from India highlighted the lack of sufficient evidence on cost effectiveness of mHealth interventions [21]. In order to fill this gap in evidence base, we undertook a study to evaluate the impact of ReMiND program on utilization of MNCH services & its cost effectiveness. In this paper, we specifically report the cost of ReMiND program in district Kaushambi.Also, we estimated the scale up cost of this program in Uttar Pradesh state which is relevant from the fiscal planning point of view.

## Methods

### Study setting & background

The ReMiND program was undertaken in district Kaushambi which is one of the high priority districts of Uttar Pradesh. Ninety two percent of its 1.6 million population resides in rural areas [22]. The female literacy rate of district is 52.7% in contrast to state average of 63.9% [3]. The district MMR of 283 deaths per 100,000 live births, was 25 and 105 points higher than the state and national averages respectively [3]. The coverage of full antenatal care checkups; haemoglobin and ultrasound test during routine ANC of pregnant women were 5%, 11.3% & 10.7% respectively [3]. Nearly 60% women in the district delivered in health facilities, while 46.9% children had full course of primary immunization during infancy [3].

To improve these maternal and child health indicators, a mHealth program named ReMiND was introduced in two community development blocks namely, Manjhanpur and Mooratganj, of district Kaushambi in year 2012. It resulted from collaborative work of two Non- Government Organizations (NGOs) i.e. Catholic Relief Services (CRS) & Vatsalya; and a social technology innovator, Dimagi. The main focus of the program was to improve the counselling skills of ASHA workers with the help of mHealth application. The detailed description of mHealth application under ReMiND program, its study setting, objectives and methodology are available as a published literature [23].

The preplanning phase of the program started with ten ASHA workers who were trained for piloting an early pregnancy checklist in March 2011. Subsequently, 111 ASHAs from Manjhanpur block were trained in August 2012 followed by training of 148 ASHAs of Mooratganj block in March 2013. These 259 ASHAs were provided Java based phones and trained on use of mobile application to register and counsel women for various MNCH services. The application had a built-in algorithm in the form of five modules which were used to advice and counsel registered women on the complete continuum of care; right from registration and antenatal care to, postnatal & newborn care as per individualized needs and requirements [24]. The data entered by ASHAs were received at Dimagi’s online cloud server. Commcare is an open source, mobile platform that enables non-technical users to build mobile applications for their frontline programs across numerous sectors reducing the need for paper based data collection. The data in variety of formats like multiple choice questions, dates, images, videos, global positioning system (GPS) coordinates can be collected through simple mobile interfaces. The data can be collected in offline mode and is sent automatically to the server on regaining internet connectivity. Thus, it can be widely used in rural areas of India which may not have good internet connectivity. Data about pregnant women were restricted and were only accessible to few authenticated users from the program with valid username and password. The data were de-identified before analysis and no reports generated by Dimagi using commcare included information about individual pregnant women. The ASHA supervisors, also called Sector facilitators, used this data to monitor the timeliness and frequency of antenatal and postnatal home care visits by ASHAs to discuss their performance in monthly meetings at block level [24].

### Data collection

Economic costing was done from the health system and patient’s perspectives to estimate the annual and per-capita cost of implementing the ReMiND program. The cost of resources spent under three time heads were obtained – preplanning phase extending from October 2010 to March 2012, start-up phase from April 2012 to July 2012, and implementation phase from August 2012 to August 2015.

We collected data from all the agencies involved in the program namely, i.e. CRS, Vatsalya, Dimagi and health department of district Kaushambi from May to October, 2015. Cost data was collected from different sources such as financial records, program budget, audit reports, agreements, etc. Data was collected from country office of Dimagi & CRS in New Delhi, state office of CRS in Lucknow and health department in district Kaushambi for health systems costs. Since CRS funded Vatsalya for implementing ReMiND program, the records for latter’s expenses were also obtained from CRS. The details about the expenditure on development and maintenance of the software were obtained from Dimagi. Maintenance cost of application included any technical assistance from the designers of the application including assistance on bug fixing and update. Along with this, costs on housing, serving, and maintaining files on the cloud, also called ‘hosting service charge’, were also obtained from Dimagi. All the cost data were obtained in US dollars, which were converted to Indian Rupees using exchange rates for the respective years. Research costs for two evaluations done during the program namely; baseline study in 2012–13 and mid-term evaluations in 2014 were excluded from the analysis.

### Intervention costing methodology

The present analysis determines the cost of ReMiND program from both the health system and patient’s perspective separately. The health system costs were stratified based on the agencies which incurred the cost (i.e. developmental partners & government health system) and nature of cost (start-up capital cost and recurrent cost). The recurrent cost in turn is stratified into cost on program implementation by development partners, cost of monitoring & supervision by government officials and the cost of providing services as a result of increased demand in public health system. The scale-up costs were estimated from the health system perspective alone. In addition to the health system cost, we also measured out of pocket expenditures (OOPE) on doctor or nurse fee, medicines, diagnostic tests, medical or surgical procedures, transportation and boarding/lodging which is called as ‘cost from patient’s perspective’. We measured OOPE incurred by households in seeking health care during pregnancy, delivery and post delivery period from a household survey [14].

### Start-up capital cost

We included two cost heads i.e. start-up capital cost and recurrent implementation cost. Start-up costs included all the capital costs incurred during the initial preplanning phase and start-up phase. Technically, any cost whose inputs may last for more than one year was considered as capital cost [25]. This was categorised as costs levied on modules’ development and piloting, development and maintenance of software, equipment cost, mobile phones and overhead costs. Apart from these costs; pre-planning meetings, trainings of ASHAs & their supervisors and translation of modules into local dialect were also taken as capital costs because the effect of these inputs were likely to last for the life of the program.

All the capital costs were apportioned in terms of their time value devoted in ReMiND program out of all the programs running simultaneously by the implementing agencies. The program was piloted in two blocks but it was designed such that it could be launched in 821 blocks of Uttar Pradesh. Accordingly, we apportioned the entire start up expenditure for the two blocks of district Kaushambi where intervention was actually piloted. After obtaining the apportioned value, costs in dollars were converted to Indian National Rupee (INR) by applying the dollar conversion rates given by the US Internal Revenue Service for a particular year of purchase of equipment [26]. The converted rates were then inflated from the year of purchase to the current value of product in 2015 by applying Consumer Price Index in India [27]. These inflated values of capital were then annualized as per the average life of utilization of the product at a discount rate of 3% [28]. The annual maintenance rates for capital items as given by the implementing agency were used in calculations.

The average life of software was assumed to be 12 years as the cost incurred on software may last either till strategies of the program remained the same; or if there was a change in technology itself. We chose former based on expert consultation as it is unlikely that any drastic paradigm change may happen in the content of the programme for another 12 years. Further, any change in technology would have had only marginal effect on costs if it requires any revision. The hosting charges were annualized for 3 years as these charges would be revised and added again like recurrent cost if the program extends beyond three years. For mobile phones, laptops, furniture and vehicle, the average life were taken as 3.5 years, 5 years, 6 and 10 years respectively as per the policy of the implementing agencies.

### Recurrent implementation cost

The implementation costs included recurrent costs which were required to sustain the intervention. All the recurrent costs were categorized as cost on human resource, travel, ASHA data/internet usage charges for mobile application, utilities like office rent, electricity, telephone bills & internet bills and stationary & printing. Other program support expenses at state and national office like the office rent and salaries were also accounted. Shared costs were apportioned as per the time value devoted by that given resource in performing a particular activity. The office rents were spent at three levels i.e. at district, state and country level. At the district level, entire rent were spent on the activities related to ReMiND program only and hence no apportioning was required. However, at the state and national levels, multiple programs were being handled simultaneously. Therefore, office rent was apportioned in proportion of floor area occupied by the staff involved in ReMiND program to the total office staff. The travel costs in form of vehicle or fuel for carrying out planning and supervisory activities during the start-up and implementation phase were dedicated to ReMiND program only as no other program was being implemented concurrently at the block levels.

For the recurrent costs, we converted the expenses levied by the partner agencies over 37 months i.e. from Aug 2012 to August 2015, into average annual expense. For converting US dollars into Indian rupees, the average dollar rate for these three years was taken as 1 US $ = INR 58.84.

### Government health system program cost

Apart from costs spent by implementing agencies, additional costs incurred by district health system were also estimated. It included three components – first, health system cost on monitoring and supervision of ASHA activities under ReMiND program. It includes the time value of the personnel involved either in their supervision through household visits, interactions with ASHA workers or indirectly via review meetings; second, incremental cost of service delivery due to increased utilization of MNCH services as the result of given intervention and third, incremental effect of intervention on ASHAs payment in terms of incentives. The direct supervisory costs were calculated considering that supervisors spend 13% of their total time in monitoring and supervisory activities, as mentioned in another study conducted in developing country [29].

For program implementation, the extra cost of monthly meetings at the block levels, quarterly meetings at district level and bi-annual meetings at the state level were calculated by apportioning the time devoted by government officials in meetings for review of ASHAs’ performance under the program. The effect of ReMiND program on overall utilization of MNCH services was evaluated using a quasi-experimental design with a difference in difference analysis [14]. The unit costs for these services were obtained from economic evidence available in the Indian context [30–32].

Economic implications of changes in service utilization in the intervention and control blocks in terms of the performance based incentives for ASHA workers were evaluated using data from health management information system (HMIS) and financial records as obtained from the office of Chief Medical Officer of the district for year 2014–15 [23].

### Unit costs

For calculation of unit costs, the population under the intervention area (387,030) was used as denominator for per capita calculation. For cost per pregnant woman, the number of women registered under mHealth was used as denominator. A total of 28,169 women have been registered under ReMiND program since its inception (over three years) as per data provided by Management Information System of the implementing agencies, so the annual number of beneficiaries was taken as 9390.

### Scale up costs of ReMiND program for Uttar Pradesh state

The intervention was implemented on pilot basis in two blocks of Uttar Pradesh state. Subsequently the estimates on the cost of implementation of ReMiND program in two blocks were used to estimate the scale up cost of this program, if the government of UP state decided to roll out intervention in the entire 821 blocks in the state. The scale up costs of mHealth intervention in entire state included costs on purchase of mobile phones, internet usage, trainings of ASHAs & their supervisors for use of application and health system costs on review meetings in the entire state. The scale up cost also implicitly included the costs of ASHAs performance based incentives. For scale up, two case scenarios were assumed. First, if current available human resource in the health system could be utilised for monitoring and supervision of this intervention. In UP, Block Community Managers (BCM) and Health Education Officers (HEO) are employed at block and district levels respectively to coordinate and supervise ASHAs’ performance. These are regular salaried staff unlike ASHAs whose remunerations were incentive based. In second scenario, a separate supervisory cadre like sector facilitators in ReMiND program were assumed at block level.

At sub-block level also, one ASHA facilitator was employed for monitoring and supervision of 20 ASHAs as guided by the National Health Mission [33], and the same pattern was followed by the implementing agencies. These were promoted ASHAs and did not receive any extra incentives for holding the position. Supervisory training costs of these ASHA facilitators were also included in the scale up cost.

As there was dearth of data on time value spent by different supervisors on various activities in our study and Indian literature [12], we assumed the time apportioned on supervisory and monitoring activities as 13% of total activity time based on findings from a similar study [29]. Though as per directions given by MoHFW, a block ASHA facilitator shall spend 20 days in field in a month [34].

From the perspective of UP state, there would be no requirement to change the content of the software or the built in audio-visual support as the same dialect could be understood in the entire state. Therefore, the start-up capital costs which included the costs on development of software, preplanning meetings, initial translation of modules were not included in scale up cost**.** Dimagi Inc. developed the software and provided free of cost server support services to first 50 beneficiaries and thereafter charged US$ 1 per year per beneficiary. Therefore, 5 million US$ have to be spent for 5 million (50 lakh) pregnant women in Uttar Pradesh as scale up hosting server charges. Table 1 describes the different types of costs calculated in the study.

**Table 1:** Various types of cost with the data sources and methodology

Area	Type of cost	Type of Data collected	Source of data	Method of data collection
At two block levels	Start up/Capital cost	Cost on Module development, translation & piloting. Development of software, Equipment and mobile cost, overheads. Preplanning meetings, ASHA trainings	Official documents of CRS, Vatsalya & Dimagi. Annual budget reports, bills receipts, Personal Interviews	Primary data collection. Time value contributed over the average age of the program
	Implementation cost	Salaries, travel, ASHA internet usage, utilities like office rent, electricity, telephone bills, stationary and printing. Program support cost at state and national office	Official documents like bills, annual budget reports, Personal interview	Primary data collection. Time apportioned as per the time value spent/utilized on the activities related only to ReMiND program
	Health system cost	Health system cost on program implementation. Incremental cost of service delivery due to increased utilization of MNCH services. unit cost for per service utilization) Incremental effect of intervention on ASHA payment.	Financial documents on ASHA incentives from Chief Medical officer’s Office, Personal Interviews, Observations during meetings at block, district and state level. Literature review for unit cost per service utilization	Primary data collection. Apportioning of the salaries of the officials as per the time spent on attending meetings at block, district and state level.
	Costs from Patient perspective	Out of pocket expenditure(OOPE) incurred by the households in seeking health care services for various maternal, newborn and child illnesses	OOPE were obtained from the Impact assessment study carried out n two intervention blocks and two control blocks.	Primary data collection
	Annual Program cost	It is sum of startup cost and implementation cost	–	Derived indicator
	Overall annual unit cost per pregnant women	Annual programmatic cost divided by average annual number of women registered under ReMiND	Management information system (MIS) data from CRS on number of registered women	Derived indicator
	Unit health system cost per pregnant women	Annual health system cost divided by average annual number of women registered under ReMiND	MIS data from CRS	Derived indicator
At state level –Uttar Pradesh	Scale up cost	Number of ASHAs in entire state, training cost of ASHA and supervisors, mobile phone cost, hosting service charges, health system cost	NRHM website, Observations, Calculations from available data	Unit costs of pilot at two blocks were expanded to 821 blocks in Uttar Pradesh state.
	Per capita scale up cost	Total scale up cost divided by Total population of state	Census 2011	Derived indicator
	Per pregnant women scale up cost	Total scale up cost in Uttar Pradesh divided by average number of pregnant women in the state in an year	Crude Birth Rate	Derived indictors

*ASHA*- Accredited Social Health Activist, *CRS*-Catholic Relief Services, *MNCH*- Maternal, Newborn and Child Health, *OOP*- Out of Pocket Expenditure, *ReMiND*- Reducing Maternal and Newborn Deaths

### Out of pocket expenditures (Patient’s perspective)

**Fig. 1:**
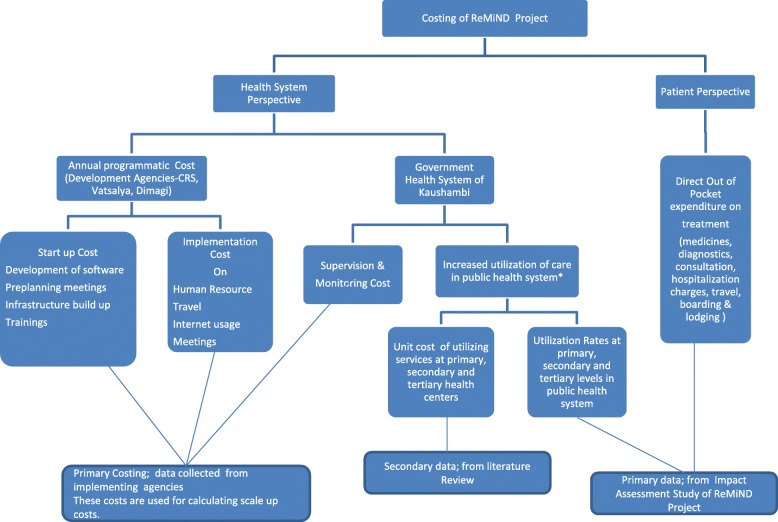
Conceptual framework for costing process of the ReMiND program. The flow chart describes the procedural detail of costing ReMiND program. The costing was undertaken from both the health system and societal perspectives

A patient’s perspective is important to consider in costing studies as it includes expenditures borne by households in form of out of pocket expenditures (OOPE) for seeking health care. Aiming patient’s perspective helps in adopting policies that intend to provide financial risk protection against OOPE and minimize the losses to households. The data on mean OOP for utilization of specific MNCH services was obtained from a household survey undertaken to evaluate impact of ReMiND and its cost effectiveness [14]. The detailed information about the household survey and its methodology are described in details in our protocol paper and impact assessment paper [14, 23]. The household questionnaire included questions to assess any cost borne by patients and their families for treatment, consultation from doctors, purchase of drugs and consumables, travel, lodging & boarding, or any money spent elsewhere. Figure 1 illustrates the conceptual framework of costing process of ReMiND program explaining both health system perspective and patient perspective.

### Sensitivity analysis

The sensitivity analysis was carried out to understand the effect of variation in input costs on both the base case estimates i.e. annual cost of ReMiND program in two blocks of district Kaushambi and the scale up cost in entire state of Uttar Pradesh. Various case scenarios were considered. The choice of upper and lower bounds for the life of the mHealth application (5 to 15 years) was based on an extensive consultation with the state program managers and development partners. The choice of percentage variation in input prices was based on review of the costing studies done in India [35–37]. It was assumed that the mobile phone and internet data charges may fluctuate to 20%. The cost of training costs of ASHAs and supervisors were fluctuated 10% and 20% respectively; trainings for latter were done in private costlier locations, while a reduction in cost can be achieved if the same were done in Government set-up and monitoring activity was increased to 50% of total time.

A review of economic evaluation studies from India, reported the use of 3%, 3.5%, 8% and 10% as the rates to discount future costs [21]. Further, the WHO-CHOICE and reference case developed by IDSi-BMGF for low and middle income countries recommends use of 3% discount rate [38, 39]. In view of this we used 3% discount rate in the base scenario. Further, since the inflation rate in India is relatively higher than most of the other developing countries [40], a discount rate below 3% was not justifiable. The other most commonly used discount rate for economic costing among developing countries are 5% and 8%. So, we varied the discount rate to 5% and 8%. The company has recently revised its hosting user charges from US$ 1 per beneficiary (pregnant women) per year to US$ 2 per ASHA worker (using mHealth application in phone) per month. In view of this, we undertook a scenario analysis during our scale up cost estimation for UP state to assess the overall and unit cost of ReMiND program in UP with changes in the hosting charges. The results of sensitivity analysis were presented in the form of tornado diagram. Sensit 1.51 software was used for the analysis.

### Ethics and consent statement

The Institute Ethics Committee of the Post Graduate Institute of Medical Sciences (PGIMER), Chandigarh provided the ethical clearance for the study vide letter no. ‘Program No IEC-01/2015-108’. Administrative approval was obtained from government health authorities of Uttar Pradesh state and Kaushambi district for obtaining health facility level data. In addition, written consent was obtained from all the participants who were interviewed in the study for obtaining the out of pocket expenditures and health system costs.

## Results

### Annual health system costs

**Fig. 2:**
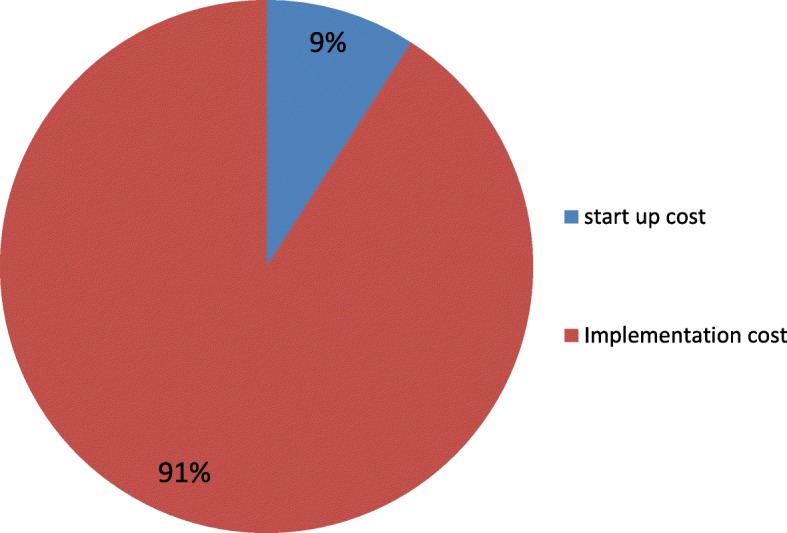
Proportional distribution of total expenditure on ReMiND program from 2011 to 2015. The figure shows the proportional distribution of start up cost and implementation cost in the total expenditure of ReMiND program

The annual cost for rolling out ReMiND program in two blocks of district Kaushambi was INR 12.1 million (US $191,894). Out of this, start-up cost and implementation cost contributed 9% and 91% of the total annual cost respectively. Government health system contributed 4.8% of the total implementation cost. The unit costs of implementing ReMiND program were INR 31.4 (US $ 0.49) per capita and INR 1294 (US $ 20.5) per registered pregnant women. Figure 2 shows the proportional distribution of total expenditure on ReMiND program in district Kaushambi. An additional file provides detailed description of start-up and implementation costs contributing to annual intervention cost for ReMiND (see Additional file 1).

### Start-up capital cost

**Fig. 3:**
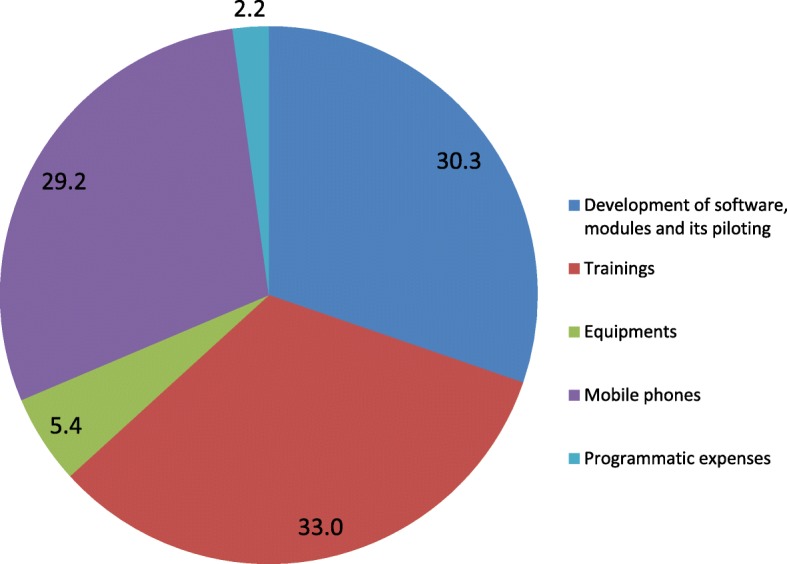
Proportional distribution of start up costs of ReMiND program in intervention area of district Kaushambi. The figure shows the proportional contribution of different start up costs in the total start up cost of ReMiND program

The start-up cost for the ReMiND program was INR 1.1 million ($17,526). A major portion of this cost was for training of ASHAs and their supervisors (33%) followed by development of software & modules and its piloting (30.3%), mobile phones (29.2%), equipment (5.4%) and programmatic expenses (2.2%). Figure 3 shows the proportional distribution of start-up costs of ReMiND program in district Kaushambi.

### Recurrent implementation cost

**Fig. 4:**
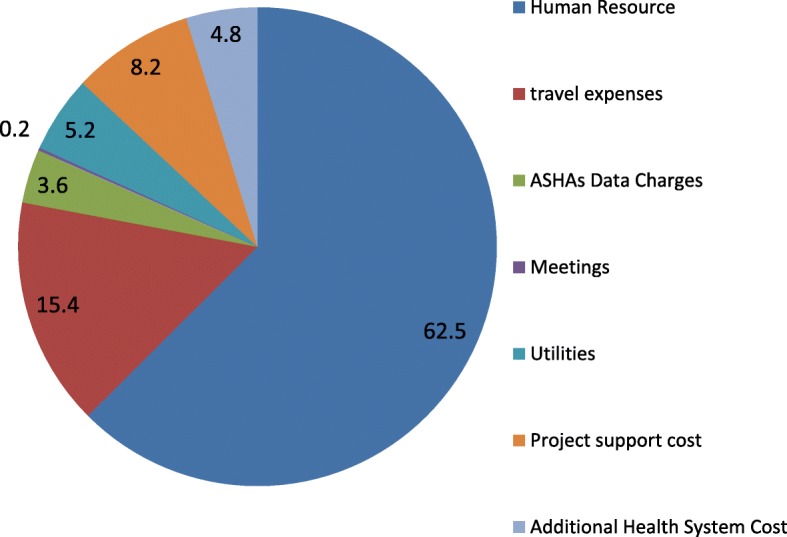
Proportional distribution of annual cost of implementation of ReMiND program in district Kaushambi. The figure shows the proportional contribution of different recurrent costs in the annual implementation cost of ReMiND program

The annual implementation cost of mHealth intervention in two blocks of Uttar Pradesh was INR 11 million (US $174,368). The costs were predominantly constituted by human resources (62.5%), followed by travel (15.4%), program support cost at national and state office of CRS (8.2%), utilities (5.2%), internet use (3.6%) and health system programme support cost (4.8%) (Fig. 4).

### Government health system program cost

The cost of monitoring the intervention from the government health system as during the review meetings was estimated to be INR 94,750 (US $ 1496.8), INR 3133 (US $ 49.5) and INR 2230 (US $ 35.2) per year at block, district and state levels respectively. The costs included apportioned time value of government officials for supervisory activities, with small contribution from travel and overheads.

We estimated that health system in intervention area had to spend INR 430,582 (US $6802) in order to cater the increased coverage of MNCH services attributable to the intervention. The incremental cost borne by the health system during implementation of ReMiND program was INR 39.9 ($ 0.63) per pregnant woman. This cost included the additional cost borne by government on monitoring and supervision of the program; increased utilization of MNCH services by pregnant and lactating mothers and increased performance based incentives to ASHAs as a result of ReMiND program. However, there was no increase in ASHA payments, hence the incremental health system cost represents the former two, i.e., cost of monitoring & supervision and cost of increase in service utilization. The detailed description of the start-up and implementation costs is given in the Additional file 1.

### Out of pocket expenditure (Patient’s perspective)

As discussed in methodology, to calculate cost of ReMiND program from patient perspective, the proportion of people bearing OOP expenditure for seeking various MNCH services and the mean OOP expenditure at various levels of health centres in both public and private sector were taken from the Impact Assessment study [14]. It was estimated that the incremental out of pocket expenditure for seeking various health care services in the intervention block was INR 15 million (US $ 240,333). The detailed out of pocket expenditures are shown in Additional file 2.

### Scale up costs

In first case scenario where intervention has to be scaled up from two blocks in Kaushambi to 821 blocks in state using the existing human resources for monitoring and supervision, we estimated the scale-up would cost around INR 876 million (US$ 13.8 million). The unit cost of scale up in Uttar Pradesh state would be INR 4.39 (US $0.07) per capita and INR 175.3 (US $2.77) per pregnant women. In second case scenario, additional human resource was assumed to be recruited for monitoring of ASHAs in every block of state. In this case, government has to spend around INR 993 million (US $15.7 million) with INR 4.97 (US $ 0.08) as per capita unit cost and INR 198.8 (US $ 3.14) as unit cost per pregnant woman (Refer Table 2). ‘An additional file describes the details of scale up costs (see Additional file 3)’.

**Table 2:** Summary of scale up costs of mHealth intervention in Uttar Pradesh in two case scenarios

Cost Description	Scenario1 When currently employed staff is used for supervisory activities INR (US$)	Scenario 2 When additional human resource is employed at the level of blocks INR (US$)
Annual cost of scale up	87,63,69,067 (13,844,693)	99,38,73,262 (15,700,999)
Annual cost per beneficiary	175 (2.77)	198.8 (3.14)
Annual cost per capita	4.39 (0.07)	4.97 (0.08)

### Sensitivity analysis

Sensitivity analysis was conducted to estimate costs in various alternate scenarios. Firstly, the total annual cost was estimated assuming that the development of software was done for two blocks of Kaushambi only. In this case, the start-up cost would be INR 4.6 million (USD 73,367) which is 30% of the total annual intervention cost of INR 15 million (USD 247,734). The incremental costs of intervention would be INR 1670 (USD 26.4) per pregnant woman and INR 40.52 (USD 0.64) per capita.

Secondly, the discount rates were varied to 5% and 8%, and the average life of program was taken as 5 and 15 years. On applying the discount rate of 5%, the incremental costs would be INR 1302 (USD 20.6) per pregnant woman and INR 31.60 (USD 0.49) per capita. At 8% discount rate, the incremental cost would be INR 1316 (USD 20.8) per pregnant woman and 31.93 (USD 0.50) per capita.

On assuming the life of programs to be 5 and 15 years, the incremental costs per pregnant woman would change to INR 1338 (USD 21.14) and 1287 (USD 20.34) respectively while per capita costs would be 32.5 (USD 0.51) and 31.23 (USD 0.49) respectively.

**Fig. 5:**
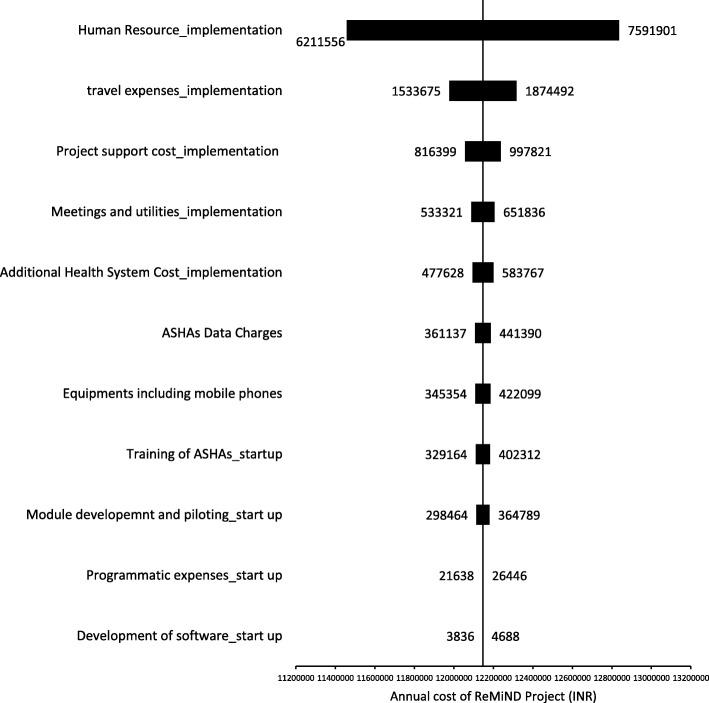
Tornado diagram illustrates the sensitivity analysis for various input factors on the annual cost (INR) of ReMiND program. The figure shows the sensitivity analysis with the help of tornado diagram to show the effect of variation in different input factors on total annual cost of ReMiND program in two blocks of Kaushambi district

**Fig. 6:**
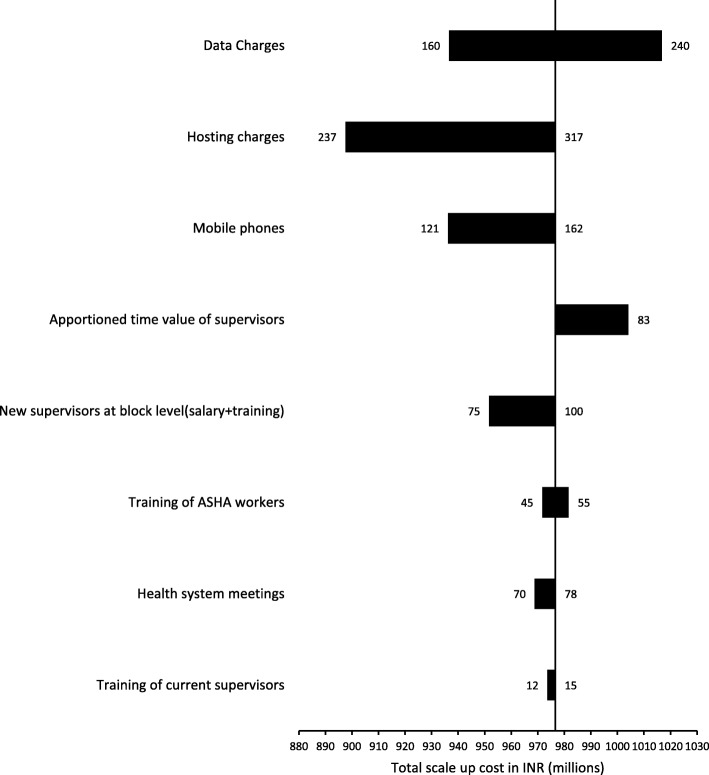
Tornado diagram illustrates the sensitivity analysis for various input factors on the scale up cost (INR) of ReMiND program in entire Uttar Pradesh. The figure shows the sensitivity analysis with the help of tornado diagram to show the effect of variation in different input factors on the total scale up cost of ReMiND program in entire Uttar Pradesh state

The tornado diagrams in Figs. 5 and 6 are the graphical representation of effect of multiple univariate input variables (represented as horizontal bars on y-axis) on the outcome variable (x-axis) i.e. annual cost of ReMiND program in two blocks of Kaushambi district and total cost of scale up of ReMiND project at the level of UP state respectively. The values shown along the both sides of black bars are range (lower and upper side) considered for varying a particular cost input parameter. More is the width of black bar, more sensitive is the outcome variable to change in input parameter. The vertical line between the black horizontal bars shows the base case results. The tornado diagram in Fig. 5 shows that salaries (human resource cost) presents the highest uncertainty and maximum impact on the total annual cost of ReMiND program (90.7% swing) followed by travel cost (5.5% swing) while start up costs like training of ASHAs (0.3% swing), purchase of equipments (0.3% swing) and development of the software (0.0%) had little influence.

Figure 6 showed that the scale up of ReMiND intervention in state of Uttar Pradesh was most sensitive to the variations in the mobile internet data charges followed by hosting charges and purchase of mobile phones. Refer Table S4A and B in ‘Additional file 4’ which presented the base cost parameter values for different input variables with their upper and lower limits for both case scenarios i.e. program cost in two blocks of Kaushambi district and scale up cost in Uttar Pradesh state.

With the revised hosting charges at the rate of US$ 2 per ASHA worker per month, UP state will have to spend INR 559 million (US $ 8.8 million) per year. The per capita costs of ReMiND program in such a scenario will be INR 2.80 (US $ 0.04) per capita and INR 111.9 (US $ 1.76) per pregnant woman.

## Discussion

Use of technology in health and other sectors has been promoted by GOI from the highest level [41]. Several other policy discourses have encouraged the application of mHealth. The High-Level Expert Group on Universal Health Coverage called for harnessing technology for promoting utilization of services [42]. Small to medium scale pilot interventions have been initiated in a variety of geographic settings in India involving a diverse range of health services such as maternal and child health to non-communicable diseases [43]. Various studies from elsewhere have also shown that the application of mHealth results in better delivery of health education, increased awareness & improved uptake of preventive as well as curative services [16, 44–46].

In Indian context, the introduction of ASHA under the National Health Mission holds strong potential for generating demand for health services [34]. However, several evaluations have shown that the knowledge and skills of ASHA workers to counsel pregnant women for their health care needs during pregnancy and nursing babies requires strengthening [12]. In light of this, use of mobile technology for improving the quality of counselling can serve as a major strategy. This can further improve knowledge and awareness among communities which may directly augment demand for services. However, it is important to understand the feasibility and financial implications of implementating this intervention as part of routine care. Hence our economic analysis holds a significant value for the policy makers in order to estimate the economic implications for implementing and scaling up of such mHealth interventions. The researchers can also use costing studies for carrying out cost effectiveness analysis of such mHealth interventions.

Our study showed that the introduction of mHealth intervention costs INR 31.4 (US $ 0.49) per capita and INR 1294 (US $ 20.5) per pregnant woman registered. The unit costs for scale up within Uttar Pradesh state were INR 4.39 ($0.07) per capita and 175.3($ 2.77) per pregnant women. The overall annual scale up cost for Uttar Pradesh to implement the same intervention in the entire state would be at least INR 876 million (US $ 13.8 million) if no additional human resource were employed for the program monitoring and support. As per our knowledge, this is the first cost outcome analysis targeting MNCH services in India [44].

### Fiscal sustainability

From the point of state budget, the total annual money allotted for reproductive & child health (RCH), maternal health (MH) and ASHA incentives for year 2015–16 in Uttar Pradesh were INR 14.6 billion, 6.99 billion and 1.22 billion respectively [47]. The introduction of mHealth intervention and its scale up in the entire state of Uttar Pradesh will represent 6.0%, 12.5% and 71.7% share in the total budget for RCH, MH and ASHA incentive program respectively. It is important to note, this scale up amount does not include introduction of any new cadre of supervisors, and rather considers training the already available cadre for supervision. As per the guidelines of Financial Management Group of National Health Mission-India’s flagship health program, it is recommended to increase the budget of high priority districts by 10–15% every year [48]. Considering the cost for scale up of ReMiND intervention to be 6% of the total budget allotted to ‘Maternal and child health’ line item under the NRHM budget of UP state, the intervention appears financially sustainable.

The World Health Report 2005 published a working paper series on scaling up of maternal and newborn interventions to reach universal health coverage by 2015. It estimated that for delivering the whole package of MNCH care (with 95% coverage) for countries in Health System Constraint Category 2 –where India fits – has an incremental cost of USD 1.53 per capita. On inflating this value to the current value of dollar by applying CPI rates in India, the current incremental cost would be USD 2.35 (INR 149.3). The WHO benefit package comprises of universal provision of comprehensive maternal, newborn and child health care services [49]. Our study shows that scale-up of mHealth in UP state will incur an incremental cost of INR 4.77 (USD0.07) per capita, which is 3% of the total incremental value proposed by WHO for achieving universal coverage of MNCH services. This again shows that the scale-up of mHealth is sustainable from fiscal viewpoint.

### Policy implications

The need to introduce a service or intervention in the benefit package of care is usually justified based on the burden of health problem which it addresses. However, the amenable burden or the reduction in the health problem which the intervention can potentially bring about is usually not considered. In light of this it is also important to understand that such mHealth application for RMNCH would thus be more useful in weaker states where the reasons for poor coverage are linked more with the demand for service. For example, the Coverage Evaluation Survey reported that poorer states such as Bihar and Uttar Pradesh have low rates of RMNCH service utilization, a large portion of which is explained by lack of awareness and knowledge about the importance of services [36]. The findings from our impact assessment study suggested an increase in knowledge and thereby utilization of MNCH services after introduction of ReMiND program [14]. Hence, such an intervention which aims at improving counselling of ASHAs, which translates into improved knowledge of community and its demand for health services, is likely to be more beneficial in these states.

Also, ReMiND intervention and its scale up appear feasible from the financial and programmatic perspectives. Currently, a quarter of Indian population who could not access healthcare cited large distances from health facilities as the reason for not being able to access health care. In such situations, mHealth can offer a useful solution. Moreover, the increased penetration of mobile use in the rural areas as well as by community health workers makes it a feasible strategy. Second, mHealth can be used as platform for strengthening programs other than RMNCHA, which are implemented through community health workers. The Government of India’s recently announced ambitious scheme of Ayushman Bharat which envisions the transformation of the existing sub-centres as Health and Wellness centres. The proposed health and wellness centres clearly outline a significant role of information technology and digital records for improving service delivery. Further, increased and timely utilization of services at these centres for primary care may decrease out of pocket expenditures on tertiary care. Global evidence suggest that mHealth solutions could lower the total annual per capita healthcare expenditure as it reduced in Brazil and Mexico by 20% and 25% respectively [50].

Also, the scale up cost of ReMiND program in UP would cost INR 993 million (US $15.7 million) if additional supervisors were to be recruited in each block for monitoring as was structured in ReMiND program while the cost would reduce to INR 876 million (US$ 13.8 million) if already employed staff was used for supervision. Dimagi Inc. charged US$ 1 per beneficiary per year as web hosting charges which has now changed to US$ 2 per ASHA worker (using mHealth application in phone) per month. This change in hosting charges would decrease the total scale up cost from INR 876.3 million to 559.8 million. Thus, it is likely that technological advances may further reduce the cost of ReMiND program. Therefore, m health solutions are emerging as useful and sustainable option from a fiscal point of view.

The mHealth program would also impact OOPE which can be understood by the theory of change explained elsewhere in a published study protocol [23]. The introduction of mHealth intervention as a job aid for community health volunteers improved their counselling skills and hence, increased knowledge among pregnant women towards early identification of the complications. This increased the demand for use of preventive and curative healthcare services. The impact assessment analysis showed that mHealth resulted in statistically significant increase in coverage iron and foIic acid (IFA) supplementation (12.70%), identification and self-reporting of illnesses/complication during pregnancy (13.20%) and after delivery (19.5%) in the intervention area as compared to the control area. The coverage of > = 3 ANC visits, > = 2 tetanus toxoid, Full ANC, and ambulance usage also increased in the intervention area by 9.7%, 4.5%, 1% and 2.5% respectively, however, the change was statistically insignificant [14]. As far as utilization of public health facilities was concerned, while 50% of women with pregnancy related complications in intervention area sought care in public hospitals, nearly 72% women sought care in private health facilities in the control area [51]. Therefore, the increased uptake of primary and secondary health care services at initial stages of illness may result in reduction in subsequent tertiary care services thus, reducing OOPE on health care. The detailed description of the theory of change and results of impact assessment survey are provided as published literature [14, 23]. The out of pocket expenditures (OOPE) for seeking maternal, newborn and child care services in private and public health facilities in the intervention and control households in reported in the table in Additional file 2. It highlighted that the out of pocket expenditures on utilization of inpatient services for newborn and childhood illnesses were lesser in intervention areas than the control areas, there is limited data available in literature from India to assess the impact of mHealth interventions on out of pocket expenditures. However, assessment reports from other LMICs like Brazil and Mexico highlighted that mHealth could reduce OOPE by US$ 102 per mHealth user thus making healthcare more affordable [50].

### Limitations

Our study had few limitations. Firstly, we could not obtain detailed year wise breakup of the expenses during the implementation phase, i.e., August 2012 to August 2015. We assumed that the same amount of expenses happened every year and averaged the cost, which might not be the case in the real situation. Second, in order to know the present worth of the capital involved in the study, we inflated the costs from year 2012 to 2015 considering the CPI Index for calculation of inflation and 3% discount rates. There is a possibility that advancement in technology over time may have actually reduced the price of capital items such as mobile phones, software etc. While this was not considered in the base case, we did undertake a sensitivity analysis to understand the impact of this assumption.

Third, since the retrospective data was collected and many of the officials from implementing agencies had left, there was a possibility of recall bias. However, as most of the data was retrieved from records, possibility of recall bas influencing the validity of our results was relatively less.

Fourth, the scale up cost implicitly included the costs of ASHAs’ performance based incentives. However, based on our impact assessment analysis, we found that the ReMiND program increased the coverage of IFA supplementation, identification and self-reporting of illnesses/ complication during pregnancy & after delivery in the intervention area as compared to control area and none of these coverage indicators were linked to any performance based incentives paid to ASHAs [14]. Also, the data obtained from the district health office did not show any substantial difference in incentives received by ASHAs in the intervention and control areas. Therefore, the effect of ASHAs incentives on scale up cost was likely to be minimal on scale up costs.

The scale up cost analysis for Uttar Pradesh state was done from the health system perspective alone. It did not include cost for increased utilization of services because of following reasons. First, the choice of selection of two blocks was not random and specifically blocks were chosen from the worst performing high priority districts. The utilization rates of services in the other districts are likely to be better than the piloted blocks. Therefore, the effectiveness values obtained in these two blocks may not be generalized in the entire Uttar Pradesh state. Moreover, the contribution from the additional cost of increased utilization of services to overall cost is less than 3.5% in the 2 blocks where ReMiND was piloted. Thus, the effect on additional cost of utilization in entire state is expected to be even less than 3.5%. Hence, it is unlikely to make any significant difference in the scale up analysis. Also, scale-up costs were estimated in the ideal conditions without considering any bottlenecks in the implementation of programme which may deviate to some extent in the real life situations. The sudden changes like political & economic instability and introduction of newer programs may affect the program in one way or other. These uncertainties could not be accounted for in our analysis. Also, we acknowledge lack of data on time spent on supervisory and monitoring activities for which the data was taken from another study [29].

Finally, as there is a keen interest globally on the use of mobile health interventions, it is furthermore important to assess the cost- effectiveness of such interventions. Our analysis is a cost-outcome description and an attempt to study the scale-up costs to understand the fiscal challenges of scale-up. However, a full economic evaluation of ReMiND program from a societal perspective was also undertaken, the results of which has been published elsewhere [23, 52]. This economic analysis estimated the incremental cost of implementing ReMiND program per disability adjusted life year averted as a result of program’s effectiveness on improving the maternal and child health outcomes. Overall, from a health system perspective, ReMiND incurred an incremental cost of INR 12,993 (USD 205) per DALY averted. From a societal perspective, ReMiND program was a cost saving intervention [52].

## Conclusion

This study is the first cost analysis of a mHealth intervention for maternal & child health services in India. Our estimates on cost are useful for policy-makers and program managers in order to plan health programs. Overall the study findings on cost of mHealth are favourable from fiscal sustainability point of view.

## Additional files

Additional file 1: Summary table for annualized start up and implementation costs of ReMiND program in district Kaushambi. The table shows the detailed description of annual costs involved during start up and implementation phase of ReMiND program. (DOCX 44 kb)

Additional file 2: Out of pocket expenditures for seeking maternal, newborn and childhood health care in public and private sector health facilities in Kaushambi, 2015. The out of pocket expenditures were estimated as a part of household survey conducted in the intervention and control areas to assess the impact of the ReMiND program. The Table presents the out of pocket expenditures of households in seeking treatment for maternal, newborn and child illnesses in private and public facilities in two areas. (DOCX 60 kb)

Additional file 3: Scale up costs for implementation of mobile health in the entire state of Uttar Pradesh in two case scenarios. The table shows the scale up cost of implementing the ReMiND program in state of Uttar Pradesh under two case scenarios. First, if the scale up is undertaken utilizing the existing human resource. Second, if additional staff is recruited for monitoring & supervisory activities at block level. (DOCX 46 kb)

Additional file 4: **Table S4A.** showing parameter base values with lower and upper limit (in INR) for implementation of ReMiND program in two blocks of district Kaushambi in Uttar Pradesh, India. **B.** showing scale up parameter base values with lower and upper limit (in INR) for implementation of ReMiND program in entire state of Uttar Pradesh, India. The table shows the base parameter cost of input variables with their lower and upper limits for the sensitivity analysis in two case scenarios. (DOCX 63 kb)

## Abbreviations

AF: Asha Facilitator
AMR: Annual Maintenance Rates
ASHA: Accredited Social Health Activist
BCM: Block Community Manager
CEAHH: Cost Effectiveness Analysis Household Survey
CHW: Community Health Workers
CPI: Consumer Price Index
CRS: Catholic Relief Services
DCPM: District Community Process Manager
HEO: Health Education Officer
IMR: Infant Mortality Rate
INR: Indian National Rupee
M & E: Monitoring and Evaluation
MH: Maternal health
mHealth: Mobile Health
MMR: Maternal Mortality Ratio
MNCH: Maternal, Newborn and Child Health
MoHFW: Ministry of Health and Family Welfare
NGO: Non- Government Organizations
NRHM: National Rural Health Mission
OOP: Out of Pocket
RCH: Reproductive & Child health
ReMiND: Reducing Maternal and Newborn Deaths
UP: Uttar Pradesh
USD: United States Dollar
WHO: World Health Organization
