# Supplementary material for: Cost analysis of implementing mHealth intervention for maternal, newborn & child health care through community health workers: assessment of ReMIND program in Uttar Pradesh, India
Source: BMC Pregnancy Childbirth. 2018 Oct 3;18:390. doi: 10.1186/s12884-018-2019-3 (PMC6171293; doi:10.1186/s12884-018-2019-3)
Supplement: Supplementary file 1 — Summary table for annualized start up and implementation costs of ReMiND program in district Kaushambi. The table shows the detailed description of annual costs involved during start up and implementation phase of ReMiND program. (DOCX 44 kb) [file 12884_2018_2019_MOESM1_ESM.docx]

Additional File 1: Summary table for annualized start up and implementation costs of ReMiND project in district Kaushambi.

|  | **Cost Category** | **Expenditure in INR (USD)** | **%age of total expenditure** |
| --- | --- | --- | --- |
| **1.** | **Total start up cost** | **11,09,395 (17,526)** | **9.0** |
| 1.1 | Development of software | 4,262 (67.3) | 0.04 |
| 1.2 | Training of ASHAs and Supervisors | 3,65,738 (5,778) | 3.0 |
| 1.3 | Equipments | 59,599 (942) | 0.5 |
| 1.4 | Purchase of mobile phones | 3,24,127 (5,121) | 2.7 |
| 1.5 | Programmatic cost | 24,042 (379.8) | 0.2 |
| 1.6 | Development of module and piloting | 3,31,627 (5,239) | 2.7 |
| **2** | **Annual Cost of implementation** | **1,10,37,461 (174,368)** | **91** |
| 2.1 | Human Resource | 69,01,728 (109,032) | 56.8 |
| 2.2 | Travel expenses | 17,04,083 (26,921) | 14.0 |
| 2.3 | Data/Internet Charges of Mobile phones | 4,01,263 (6,339) | 3.3 |
| 2.4 | NGO Meetings | 20,774 (328) | 0.2 |
| 2.5 | Utilities* | 5,71,804 (9,033) | 4.7 |
| 2.6 | Project support cost (National &state office of CRS) | 9,07,110 (14,330) | 7.5 |
| 2.7 | Health system cost Programme support Cost | 5,30,698 (8383) | 4.3 |
| Total annual intervention cost | | 1,21,46,856 (191,894) | 100 |
| Unit cost per pregnant woman | | 1293.6 (20.5) | |
| Unit cost per capita | | 31.4 (0.49) | |
| *Utilities include recurrent cost like office rent, electricity & communication, internet expenses, printing and stationary.  Project support costs include apportioned cost of Human resource, office rent, travel made for ReMiND project at state and national level offices of implementation partners.  Additional health system cost includes extra cost on the health system due to increased utilisation of services in the intervention area as a result of effect of counselling on the care seeking patterns and utilisation of health services and on meetings held at block, district and state level for review of ASHA work under ReMiND project | | | |
