# Supplementary material for: Cost analysis of implementing mHealth intervention for maternal, newborn & child health care through community health workers: assessment of ReMIND program in Uttar Pradesh, India
Source: BMC Pregnancy Childbirth. 2018 Oct 3;18:390. doi: 10.1186/s12884-018-2019-3 (PMC6171293; doi:10.1186/s12884-018-2019-3)
Supplement: Supplementary file 2 — Out of pocket expenditures for seeking maternal, newborn and childhood health care in public and private sector health facilities in Kaushambi, 2015. The out of pocket expenditures were estimated as a part of household survey conducted in the intervention and control areas to assess the impact of the ReMiND program. The Table presents the out of pocket expenditures of households in seeking treatment for maternal, newborn and child illnesses in private and public facilities in two areas. (DOCX 60 kb) [file 12884_2018_2019_MOESM2_ESM.docx]

Additional File 2: **Out of pocket expenditures for seeking maternal, newborn and childhood health care in public and private sector health facilities in Kaushambi, 2015**

| **Indicator** | **Category** | **Intervention** (INR) | | **Control** (INR) | |
| --- | --- | --- | --- | --- | --- |
|  |  | Mean | Standard Error | Mean | Standard Error |
| Institutional Delivery | Public | 861 | 75 | 610 | 32 |
|  | Private | 16,900 | 2984 | 13,000 | 942 |
| Hospitalisation  for childhood illnesses | Public | 2,227 | 640 | 5,550 | 2,090 |
|  | Private | 3,800 | 563 | 9,975 | 2,109 |
| Hospitalization for newborn illnesses | Public | 380 | 120 | 2,357 | 2,322 |
|  | Private | 1,000 | 0 | 5,164 | 1,084 |
| OPD for childhood illnesses | Public | 661 | 78 | 1,365 | 571 |
|  | Private | 828 | 60 | 540 | 45 |
| OPD for newborn illnesses | Public | 1,399 | 504 | 283 | 162 |
|  | Private | 800 | 153 | 769 | 152 |
