# Supplementary material for: Cost analysis of implementing mHealth intervention for maternal, newborn & child health care through community health workers: assessment of ReMIND program in Uttar Pradesh, India
Source: BMC Pregnancy Childbirth. 2018 Oct 3;18:390. doi: 10.1186/s12884-018-2019-3 (PMC6171293; doi:10.1186/s12884-018-2019-3)
Supplement: Supplementary file 3 — Scale up costs for implementation of mobile health in the entire state of Uttar Pradesh in two case scenarios. The table shows the scale up cost of implementing the ReMiND program in state of Uttar Pradesh under two case scenarios. First, if the scale up is undertaken utilizing the existing human resource. Second, if additional staff is recruited for monitoring & supervisory activities at block level. (DOCX 46 kb) [file 12884_2018_2019_MOESM3_ESM.docx]

Additional File 3: Scale up costs for implementation of mobile health in the entire state of Uttar Pradesh in two case scenarios.

| **Costing Heads***  **@ Annualized unit cost/ year (INR)** | **Number of units** | | **Scenario 1: utilising the existing human resource INR (US $)** | **Scenario2: Recruiting new cadre of human resource INR (US $)** |
| --- | --- | --- | --- | --- |
| ASHA Training cost @  INR 384.0/ASHA/year | Total number of ASHAs in UP =129312 | | 49,607,076 | 49,607,076 |
| Mobile phones @  INR 1251.5/ASHA/year |  |  | 161,828,403.5 | 161,828,404 |
| Annual Data Charges  @1549.3/ASHA/year |  |  | 200,340,413.3 | 200,340,413 |
| Training of supervisor for M& E @ INR 2160/supervisor/year | 75 HEO+ 75 DCPM+ 821 BCM+ 6000ASHA facilitators  (=6971 supervisors) | | 15,054,223 | 15,054,223 |
| Additional staff –salary  @ 10,000 per month | 1 supervisor per block  =821 additional supervisors | | Not Applicable | 98,520,000 |
| Additional Staff-Training  @2160* /person/year | 821 new supervisors | | Not Applicable | 1,773,360 |
| Monitoring and supervisory cost =13% of total salary of supervisory staff | HEO=35000 pm | | 55,013,400 | 67,821,000 |
|  | DCPM=31000pm | |  |  |
|  | BCM=15000 pm | |  |  |
|  | AF=3000 pm | |  |  |
|  | New supervisor=10,000 | |  |  |
| **Expenditure on meetings for review of m-health within health system** | | | | |
| block level  @ 94,751 (scenerio1)/block  @100104 (scenario2)/block | Total blocks in UP= 821 | 77,790,513 | | 82,185,384 |
| district level  @3133.83(scenario1)/district  @3245.353(scenerio2)/district | Total districts in UP= 75 | 235,037 | | 243,401 |
| Hosting charges @1 US $/ beneficiary/year)  (1 USD=INR 63.3) | 5 million beneficiaries | 316,500,000 | | 316,500,000 |
| Total cost of scale up |  | 876,369,067( 13,844,693) | | 993,873,262 (15,700,999) |
| Unit cost per beneficiary |  | 175 (2.77) | | 198.8 (3.14) |
| Unit cost per capita |  | 4.39 (0.07) | | 4.97 (0.08) |

ASHA= Accredited Social Health Activist, AF= ASHA facilitator, BCM= Block Community Manager,

DCPM= District Community process Manager, HEO= Health Education officer,

INR= Indian National Rupee, M & E= Monitoring and Evaluation,

UP= Uttar Pradesh, USD= United States Dollar.
