# Supplementary material for: Cost analysis of implementing mHealth intervention for maternal, newborn & child health care through community health workers: assessment of ReMIND program in Uttar Pradesh, India
Source: BMC Pregnancy Childbirth. 2018 Oct 3;18:390. doi: 10.1186/s12884-018-2019-3 (PMC6171293; doi:10.1186/s12884-018-2019-3)
Supplement: Supplementary file 4 — Table S4A. showing parameter base values with lower and upper limit (in INR) for implementation of ReMiND program in two blocks of district Kaushambi in Uttar Pradesh, India. B. showing scale up parameter base values with lower and upper limit (in INR) for implementation of ReMiND program in entire state of Uttar Pradesh, India. The table shows the base parameter cost of input variables with their lower and upper limits for the sensitivity analysis in two case scenarios. (DOCX 63 kb) [file 12884_2018_2019_MOESM4_ESM.docx]

| Input Variable | Base Case | Lower Limit | Upper Limit |
| --- | --- | --- | --- |
| Recurrent implementation Cost |  |  |  |
| Human Resource | 6,901,728 | 6,211,556 | 7,591,901 |
| travel expenses | 1,704,083 | 1,533,675 | 1,874,492 |
| Project support cost | 907,110 | 816,399 | 997,821 |
| Meetings and utilities | 592,579 | 533,321 | 651,836 |
| Additional Health System Cost | 530,698 | 477,628 | 583,767 |
| ASHAs Data Charges | 401,263 | 361,137 | 441,390 |
| Start up Capital cost |  |  |  |
| Equipments including mobile phones | 383,727 | 345,354 | 422,099 |
| Training of ASHAs | 365,738 | 329,164 | 402,312 |
| Module development and piloting | 331,627 | 298,464 | 364,789 |
| Programmatic expenses | 24,042 | 21,638 | 26,446 |
| Development of software | 4,262 | 3,836 | 4,688 |

Table S4 A: Table showing parameter base values with lower and upper limit (in INR) for implementation of ReMiND program in two blocks of district Kaushambi in Uttar Pradesh, India

*The graphical representation of the data is provided in Figure 5 of the manuscript as Tornado diagram

Table S4B: Table showing scale up parameter base values with lower and upper limit (in INR) for implementation of ReMiND program in entire state of Uttar Pradesh, India

| Input Variable | Base Case | Lower Limit | Upper Limit |
| --- | --- | --- | --- |
| Data Charges | 200,340,413 | 160,272,331 | 240,408,496 |
| Hosting charges | 316,500,000 | 237,375,000 | 316,500,000 |
| Mobile phones | 161,828,404 | 121,371,303 | 161,828,404 |
| Apportioned time value of supervisors | 55,013,400 | 55,013,400 | 82,520,100 |
| New supervisors at block level(salary+training) | 100,293,360 | 75,308,688 | 100,293,360 |
| Training of ASHA workers | 49,607,076 | 44,646,368 | 54,567,784 |
| Health system meetings | 78,025,550 | 70,222,995 | 78,025,550 |
| Training of current supervisors | 15,054,223 | 12,043,378 | 15,054,223 |

*The graphical representation of this table is provided in Figure 6 of the manuscript as Tornado diagram
